# Investigating the Potential Signaling Pathways That Regulate Activation of the Novel PKC Downstream of Serotonin in *Aplysia*

**DOI:** 10.1371/journal.pone.0168411

**Published:** 2016-12-21

**Authors:** Carole A. Farah, Bryan Rourke, Unkyung Shin, Larissa Ferguson, María José Luna, Wayne S. Sossin

**Affiliations:** Department of Neurology and Neurosurgery, Montreal Neurological Institute, McGill University, Montreal, Quebec, Canada; Texas A&M University Corpus Christi, UNITED STATES

## Abstract

Activation of the novel PKC Apl II in sensory neurons by serotonin (5HT) underlies the ability of 5HT to reverse synaptic depression, but the pathway from 5HT to PKC Apl II activation remains unclear. Here we find no evidence for the *Aplysia*-specific B receptors, or for adenylate cyclase activation, to translocate fluorescently-tagged PKC Apl II. Using an anti-PKC Apl II antibody, we monitor translocation of endogenous PKC Apl II and determine the dose response for PKC Apl II translocation, both in isolated sensory neurons and sensory neurons coupled with motor neurons. Using this assay, we confirm an important role for tyrosine kinase activation in 5HT mediated PKC Apl II translocation, but rule out roles for intracellular tyrosine kinases, epidermal growth factor (EGF) receptors and Trk kinases in this response. A partial inhibition of translocation by a fibroblast growth factor (FGF)-receptor inhibitor led us to clone the *Aplysia* FGF receptor. Since a number of related receptors have been recently characterized, we use bioinformatics to define the relationship between these receptors and find a single FGF receptor orthologue in *Aplysia*. However, expression of the FGF receptor did not affect translocation or allow it in motor neurons where 5HT does not normally cause PKC Apl II translocation. These results suggest that additional receptor tyrosine kinases (RTKs) or other molecules must also be involved in translocation of PKC Apl II.

## Introduction

Protein Kinase Cs (PKCs) are Serine/Threonine kinases which are involved in many cellular processes including the synaptic plasticity that underlies learning and memory formation [1–2]. There are four families of PKC isoforms in vertebrates: the conventional or Ca^2+^-dependent PKCs (cPKCs) family which includes PKC α, β1, β2 and γ, the novel or Ca^2+^-independent PKCs (nPKCs) of the epsilon family which includes PKC ε and η the nPKCs or Ca^2+^-independent PKCs of the delta family which includes PKC δ and θ and the atypical family (aPKCs) which includes PKC ζ and ι [2]. In *Aplysia*, a model system that has been extensively used to study memory formation [3,4], there are three PKC isoforms expressed in the nervous system: the conventional PKC Apl I, the novel epsilon family member PKC Apl II and the atypical PKC Apl III [2]. In this system, the synaptic plasticity underlying simple forms of non-associative learning has been elucidated. In particular, habituation is mediated by synaptic depression between sensory neurons and motor neurons and can be produced in culture by stimulating the sensory neuron at a low frequency [5]. Dishabituation, which is produced by giving an aversive stimulus to a habituated animal leading to the loss of habituation is mediated by the reversal of depression and can be elicited in culture by applying the neurotransmitter serotonin (5HT) to previously depressed synapses [6]. Indeed, aversive stimuli cause the release of 5HT [7]. Interestingly, the cellular mechanisms underlying facilitation at naïve synapses seem to be different from those underlying facilitation at depressed synapses since the former requires PKA [8–10] whereas the latter requires the novel PKC Apl II [9,11] acting in the sensory neuron. Novel PKCs were also shown to regulate sensory neuron activity in other species. For example, the orthologue of PKC Apl II in vertebrates, PKCε, is involved in sensitization of sensory neurons in rodents [12,13], as is the orthologue of PKC Apl II in *C*. *elegans* [14].

We have previously shown that PKC Apl II translocates to the plasma membrane in response to 5HT in sensory neurons [15]. This response depends both on diacylglycerol (DAG) produced downstream of phospholipase C (PLC) activation and on phosphatidic acid (PA) produced downstream of phospholipase D (PLD) activation [16]. However, how 5HT is coupled to these downstream signalling pathways is not clear. We previously found that the *Aplysia* 5HT receptors, 5HT_2Apl_ and 5HT_7Apl,_ can couple to 5HT-mediated PKC Apl II translocation in a heterologous cell line, Sf9 cells [1]. However, the 5HT_2_ antagonist pirenperone, which blocked the response to 5HT when 5HT_2Apl_ was expressed in Sf9 cells, did not block 5HT-mediated translocation of PKC Apl II in sensory neurons, nor did it block 5HT-mediated reversal of depression [1]. Moreover, expression of 5HT_2Apl_ was not sufficient for 5HT to translocate PKC Apl II in motor neurons, where 5HT is normally not sufficient to stimulate PKC Apl II translocation [1]. While activation of PKCε in vertebrates can be downstream of cyclic adenosine monophosphate (cAMP) [13], knocking-down the 5HT receptor coupled to cAMP production, 5HT_7Apl,_ did not block the reversal of depression mediated by PKC Apl II [17]. Interestingly, the tyrosine kinase inhibitor genistein blocked both 5HT-mediated PKC Apl II translocation and reversal of depression suggesting a non-canonical mechanism for activation of PKC Apl II [1].

In the present study, we investigated alternative pathways that may lead to PKC Apl II translocation in response to 5HT. First, we used translocation of endogenous PKC Apl II to examine the dose response for PKC Apl II activation and the role of synapse formation on the dose required. Next, based on the effect of genistein, we examined a battery of more specific tyrosine kinase inhibitors and showed that of these, only the fibroblast growth factor receptor (FGFR)-1 inhibitor SU-5402 significantly inhibited 5HT-mediated translocation of PKC Apl II in sensory neurons. However, overexpressing *Aplysia* FGFR1-like receptor in isolated motor neurons was not sufficient to allow translocation, nor did it affect translocation in isolated sensory neurons. Thus, while FGFRs may play a supplementary role in PKC Apl II translocation, they do not fully explain the requirement for tyrosine kinase activation. Finally, we tested other putative 5HT receptors. We cloned B2 and B4 receptors which are closely related to serotonergic and dopaminergic receptors [1] and showed that they cannot activate PKC Apl II in response to 5HT.

## Methods

This work was approved by the MNI Animal Care and Use committee

### Constructs

**Fig 1:**
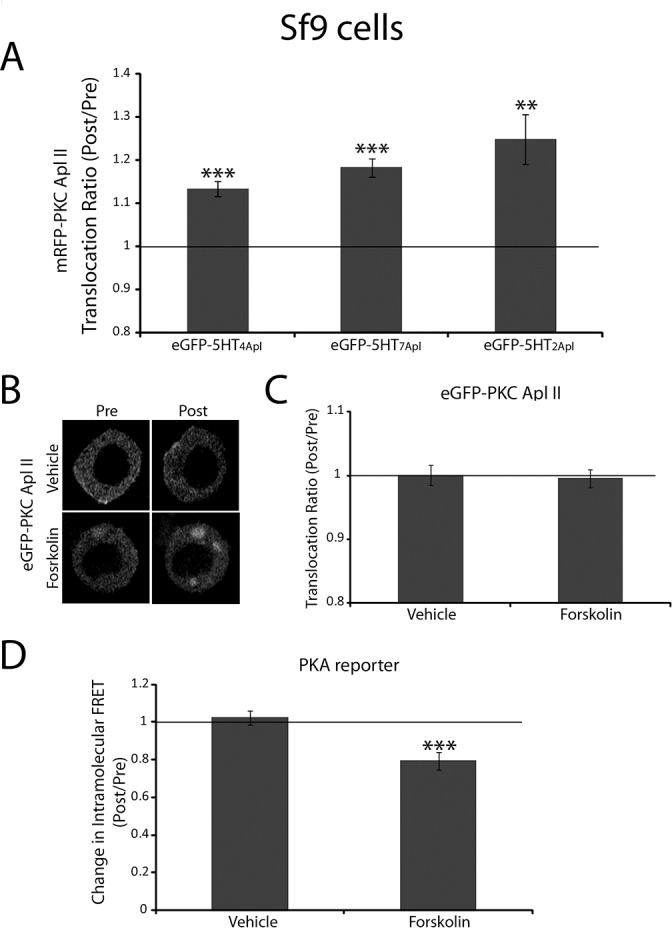
Adenylate cyclase activation does not cause translocation of PKC Apl II in response to 5HT in Sf9 cells. **A)** Sf9 cells were cotransfected with mRFP-PKC Apl II along with either eGFP-5HT_4Apl_ (n = 17), eGFP-5HT_7Apl_ (n = 15) or eGFP-5HT_2Apl_ (n = 9). Cells were treated with 5HT (10 μM) for 5 min and PKC Apl II translocation ratio (Post/Pre treatment) was quantified as described in methods. The bar graph shows the average of the translocation ratios measured at 60, 90, 120 and 150 sec after the addition of 5HT to the dish normalized to the Pre 5HT group. A paired Student’s t test was used to compare translocation post 5HT to pre 5HT for each group, ***p<0.001, **p<0.01 after correction for multiple tests with Bonferroni correction. Error bars indicate SEM. **B)** Representative confocal fluorescence images of Sf9 cells expressing eGFP-PKC Apl II before forskolin (100 μM) treatment or 6 min following forskolin (100 μM) treatment. **C)** The translocation ratio (Post/Pre) was quantified for the cells shown in B. No translocation was noted for the forskolin group (n = 12) or the vehicle group (n = 9). **D)** Change in intramolecular FRET (Post/Pre) was calculated for Sf9 cells co-expressing the regulatory subunit of PKA tagged with CFP (RII-CFP) and the catalytic subunit tagged with YFP (C-YFP) that were treated either with vehicle (n = 9) or with forskolin (100 μM; n = 10) for 4 min. cAMP production downstream of forskolin treatment and its binding to the regulatory subunits of PKA causes dissociation of the regulatory and catalytic subunits of PKA leading to a loss of FRET. A paired Student’s t test was used to compare the FRET ratio Post-treatment to Pre-treatment for each condition. Forskolin treatment caused a significant decrease of the FRET ratio, ***p<0.001. Error bars indicate SEM.

The sequence of the previously cloned B receptors was used to screen the *Aplysia* genome at NCBI and a number of hits on adjoining genomic fragments were found (Fig 1A). PCR primers were generated from all the putative receptors using diverged regions of the receptor (S1 Table) and a nervous system cDNA library was screened. All receptors were amplified from the nervous system. To clone the B2 and B4 receptors, primers were generated to clone the full length receptors B2 Forward primer AACACCTGAGATGTCTAC, B2 Reverse primer AGTCTACCGATTCATTGGCTG, B4 Forward primer AGGACAGTGACTAGTGTTAC and B4 Reverse primer AATTGCTTTCCAACGTCATGG and then these sequences were amplified with BsrG1 and KpnI sites on the primers (B2) or BsiWI and KpnI sites (B4) on the primers to insert the receptors directly into pNEX-(sen-eGFP), which contains the sensorin signal sequence followed by DNA encoding eGFP, followed by restriction sites to insert receptor sequences [1]. This allows expression of tagged receptors and avoids issues with long N-terminal domains that can lower expression of the receptors [1]. The plasmids were confirmed by sequencing.

A bioinformatic screen was performed to identify a putative FGFR sequence in *Aplysia californica*. An *Aplysia* trace search was conducted using the Basic Local Alignment Search Tool (BLAST; www.ncbi.nlm.nih.gov/blast), and it identified sequences of *Aplysia* genomic DNA that are homologous to FGFR sequences in other invertebrates, such as *Drosophila melanogaster* and *Crassostrea*. The search results were cross-referenced on the *Aplysia* Transcriptome Assembly (www.aplysiagenetools.org), and a putative sequence was obtained.

*Aplysia* FGFR receptor sequence was amplified by PCR from an *Aplysia* nervous system cDNA library in three segments since it was not possible to amplify the full-length sequence at once (primers listed in S2 Table). Each segment was subcloned into the pJET1.2 expression vector using the Thermo-Scientific CloneJET PCR cloning kit. Ap FGFR1-like segments were then transferred sequentially into the *Aplysia* expression vector, mRFP-pNEX3 using overlap extension PCR [18,19] (primers listed in S3 Table). Phusion DNA polymerase was used to PCR-amplify each segment from the pJET1.2 plasmid. The PCR products were gel-purified and used in the overlap extension PCR reaction with the mRFP-PNEX3 vector. The overlap extension PCR products were then digested with Dpn1 to destroy the parental plasmid and the remaining products used for subsequent transformation in XL10-Gold Ultracompetent Cells (Agilent).

eGFP-PKC Apl II, mRFP-PKC Apl II and eGFP-5HT_4Apl_ were previously described [1,11,15]. PKA FRET reporter [RII-cyan fluorescent protein (CFP) and Cat-yellow fluorescent protein (YFP)] has been previously described [20] and was a kind gift from Dr Manuela Zaccolo and Dr Marc Klein.

### Sf9 cell culture

Sf9 cell cultures were prepared based on a previously described method [21]. Sf9 cells (Sigma) were grown as a monolayer in 35 mm 30 glass-bottomed culture dishes (MatTek) at a concentration of 0.11x10^6^ cells per dish. Cells were grown in Grace’s media (Invitrogen) supplemented with 10% fetal bovine serum (Cansera) at 27°C. Cells were transfected 24 hrs post plating using the transfection reagent Cellfectin (Invitrogen) following the manufacturer’s instructions and the experiments were performed 48 hours later. The pNEX3 vectors were used for protein expression in Sf9 cells.

### *Aplysia* neuronal cultures

Adult *Aplysia californica* (76-100g) were purchased from the University of Miami Aplysia Resource Facility (RSMAS, FL, USA). Animals were anesthetized with an injection of 50–100 mL of 400 mM (isotonic) MgCl_2_. Abdominal and/or pleuropedal ganglia were then removed and digested in L15 medium containing 10 mg/mL Dispase II (Roche) for 20 hours at 19°C. L15 medium (Sigma) was supplemented with NaCl, 0.2 M and (in mm)–MgSO4 7H_2_O, 26; dextrose, 35; MgCl_2__6H_2_O, 27; KCl, 4.7; NaHCO_3_, 2; CaCl_2__2H_2_O, 9.7; and HEPES, 15; and the pH was adjusted to 7.4. Following digestion, siphon motor neurons (LFS) and tail sensory neurons were isolated from the abdominal and the pleural ganglia respectively and plated in L15 medium containing 50% *Aplysia* hemolymph on MatTek glass-bottomed culture dishes (MatTek). The dishes were pretreated with poly-L-lysine (molecular weight > 300 000; Sigma). Sensory neurons were manually paired with motor neurons to allow for synapse formation and cells were cultured at 19°C in a high-humidity chamber. Cultures were grown for 48–72 hours prior to the experiment. For sensory-motor neuron cocultures, the presence of functional synapses was confirmed electrophysiologically.

### Confocal microscopy in Sf9 cells and in neurons

For live-cell imaging experiments in Sf9 cells, cultures were serum starved 2 hrs prior to imaging and then imaged using a Zeiss LSM 710 confocal microscope with a 63x oil immersion lens in a temperature controlled chamber maintained at 27°C as previously described [21]. Cells were treated with 5HT (10μM) or a vehicle solution for 5 min and pictures were taken pre and post treatment. Imaging of fixed neuronal cultures was performed on a Zeiss LSM 710 confocal microscope with a 40x oil immersion lens. For both Sf9 cells and neurons, pictures of the cell bodies were taken at the middle section of the cells, with the nucleus as well defined as possible. In experiments in neurons using a pharmacological inhibitor, cells were pre-treated with various inhibitors, followed by 5HT treatment at the indicated concentration in the presence of the inhibitor.

### Quantification of PKC Apl II translocation

Quantification of PKC Apl II translocation was performed using Image J (NIH) software as previously described [16]. Briefly, the level of PKC Apl II translocation in the cell body was determined by tracing three rectangles at different locations at the plasma membrane and three rectangles at different locations in the cytosol. The width of the membrane rectangles was three to five pixels wide to minimize cytoplasmic contamination, but otherwise the size of the rectangles was not constrained. The cytosol rectangles were traced in the areas adjacent to the membrane rectangles. In live-imaging experiments in Sf9 cells and in neurons, the translocation ratio was measured as the average intensity (membrane) [Im] /average intensity (cytosol) [Ic] normalized to the degree of translocation before the addition of pharmacological agents ([Im/Ic-Post] / [Im/Ic-Pre]). In fixed neuronal cultures, the translocation ratios (Im/Ic) in the drug-treated group were normalized to the average translocation ratio (Im/Ic) in the 5HT alone group from the same experiment. This was referred to as membrane enrichment (Im/Ic) normalized to 5HT alone. For dose response experiments, a double normalization was performed. In this case, [Im/Ic-at different 5HT concentrations] was subtracted from [Im/Ic-at 0 μM 5HT] and the result normalized to [Im/Ic-at 10 μM 5HT]. Data are presented as means ± standard error of the mean (SEM).

### Chemicals and antibodies

The following chemicals were purchased from Sigma and used at the indicated concentrations and pre-treatment periods: Genistein (100 μM) for 10min, Vanadate (1 mM) for 30 min, PP1 (10 μM) for 5 min, PP2 (25 μM) for 5 min, K252a (100 nM) for 5 min, SU5402 (40 μM) for 60 min. Herbimycin A (5 μM) for 15 min and Lavendustin A (50 μM) for 15 min were purchased from Tocris. Bisindolylmaleimide (200 nM) for 30 min, was purchased from EMD. Forskolin (100 μM) was purchased from Sigma and Sf9 cells were treated with it for 6 min. The antigen and purification strategy for the anti-PKC Apl II antibody was previously described [22].

### FRET and image quantification

FRET experiments were performed as previously described [23]. Briefly, pictures of cells expressing CFP alone or YFP alone were taken to provide measurements for bleed-through subtraction. Cells expressing the CFP-PKA-YFP construct were imaged in the CFP, YFP and FRET configurations keeping exposure times constant for all groups within each experiment. FRET images were analyzed using Zeiss AxioVision software. The FRET Xia formula was used to allow for subtraction of bleed-through from the cyan and yellow channels [24]. A FRET ratio was obtained ranging from 0 to 1. A decrease in FRET ratio corresponds to activation of PKA. Images were taken pre and at different time points post treatment. The fold change in FRET signal (Post/Pre) was then calculated.

### Immunocytochemistry

Following 5HT treatment cells were fixed with a solution of 4% paraformaldehyde and 30% sucrose in phosphate buffered saline (PBS) for 30 min. Cells were then permeabilized with 0.1% Triton X-100 and 30% sucrose in PBS for 15 min followed by one wash in PBS and NH_4_Cl for 15 min, to quench free aldehydes. Cells were then treated with a blocking solution of 10% normal goat serum and 0.5% Triton X-100 in PBS for 30 min before incubating them with the PKC Apl II antibody at a dilution of 1:2000 for 1 hr. Three to four washes of PBS (5 min) were then performed followed by incubation with a secondary goat anti-rabbit FITC antibody at a dilution of 1:200 for 1 hour. Cells were washed with PBS and stored in PBS until imaging.

## Results

### Adenylate Cyclase activation is not sufficient to induce PKC Apl II translocation in Sf9 cells

We have previously shown that both diacylglycerol (DAG) produced down stream of phospholipase C (PLC) activation and phosphatidic acid (PA) produced downstream of phospholipase D (PLD) activation synergize to translocate PKC Apl II to the plasma membrane in response to serotonin [16]. However, the molecules upstream of of PLC and PLD have not been identified. In vertebrates, adenylate cyclase was shown to be important for activation of PLC and PLD, both of which are necessary for cAMP mediated translocation and activation of the novel PKCε [13]. Furthermore, overexpressing the adenylate cyclase-coupled 5HT_7Apl_ [1] or 5HT_4Apl_ (Fig 1A) receptors caused translocation of mRFP-tagged PKC Apl II in response to 5HT (10 μM) in Sf9 cells. However, removal of the adenylate cyclase-coupled 5HT_7Apl_ receptor in Aplysia neurons using siRNA does not block reversal of depression, suggesting that this is not the receptor coupled to PKC Apl II activation in sensory neurons [17]. To better understand why activation of 5HT_7Apl_ can cause translocation in Sf9 cells but not in neurons, we examined if adenylate cyclase activation was sufficient to induce translocation of PKC Apl II in Sf9 cells. We overexpressed eGFP-PKC Apl II in Sf9 cells and treated them with the cell-permeable activator of adenylate cyclase forskolin for 6 min at a concentration of 100 μM. As shown in Fig 1B and 1C, forskolin did not cause translocation of eGFP-PKC Apl II and the translocation ratio in the forskolin group was similar to that in the vehicle group. To confirm that forskolin was activating adenylate cyclase, we overexpressed a previously described FRET-based PKA reporter [20] in Sf9 cells and treated the cells with forskolin (100 μM) for 4 min. PKA is activated by cAMP produced dowstream of adenylate cyclase. Upon cAMP binding to the CFP-tagged regulatory subunits of PKA, the subunits dissociate from the YFP-tagged catalytic subunits causing a loss of FRET. As shown in Fig 1D, forskolin treatment induced a significant loss of FRET compared to vehicle treatment demonstrating activation of adenylate cyclase despite the lack of translocation of eGFP-PKC Apl II by forskolin. This result suggests that adenylate cyclase activation is not sufficient to cause translocation of PKC Apl II and thus, an unknown pathway activated by G_s_-coupled receptors in Sf9 cells is required for PKC Apl II translocation seen in these cells.

### Synapse formation lowers the serotonin requirement for translocation of endogenous PKC Apl II

**Fig 2:**
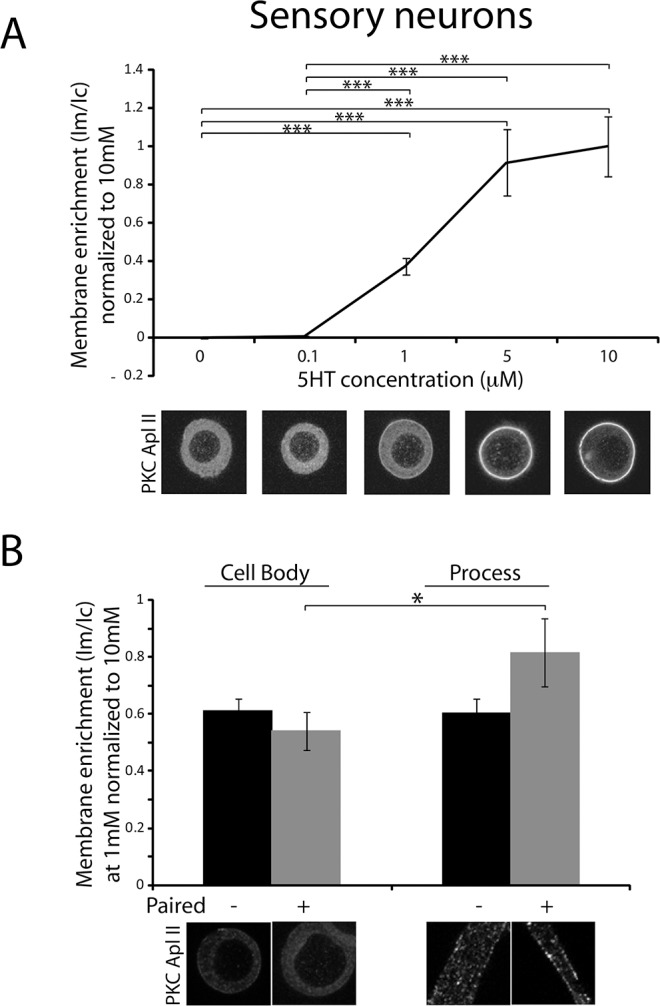
Concentration dependence of serotonin induced translocation of endogenous PKC Apl II in the cell body and the processes of isolated sensory neurons and co-cultures of sensory and motor neurons. **A)** The top panel shows the quantification of PKC Apl II membrane enrichment in isolated sensory neurons (shown in the bottom panel) treated with serotonin concentrations of 0 μM (n = 26 cells), 0.1 μM (n = 33 cells), 1 μM (n = 59 cells), 5 μM (23 cells) and 10 μM (n = 30 cells) and included results from three independent experiments. Cells were treated with 5HT for 5 min and then fixed and processed for immunocytochemistry using an anti-PKC Apl II antibody as described in methods. For each experiment, membrane enrichment ratio was subtracted from the membrane enrichment ratio in the vehicle group (5HT concentration of 0 μM) and then normalized to the membrane enrichment ratio in the 5HT (10 μM) group. A Kruskal-Wallis test was performed to compare the normalized membrane enrichment in the different groups, *** p<0.001. Error bars indicate SEM. **B)** The top panel shows the quantification of PKC Apl II membrane enrichment in the cell body and the processes of isolated sensory neurons (unpaired) or sensory neurons paired with motor neurons (shown in the bottom panel) treated with a serotonin concentration of 1 μM for 5 min (n = 26 unpaired cells and n = 6 paired cells from three independent experiments). For each group, membrane enrichment ratio was normalized to the membrane enrichment ratio in the 5HT (10 μM) group. An unpaired Student’s t test was performed to compare membrane enrichment in the processes of sensory neurons paired with motor neurons to that in the cell body, *p<0.05. Error bars indicate SEM.

PKC Apl II was shown to mediate short-term facilitation at previously depressed synapses [11]. However, this form of facilitation can be induced by serotonin concentrations as low as 1 μM in *Aplysia* ganglia [25] while activation of overexpressed fluorescently-tagged PKC Apl II is typically observed at 10 μM 5HT in cultured sensory neurons from *Aplysia* [15,16]. However, one caveat of measuring the dose response for translocation of eGFP-tagged PKC Apl II is that the overexpression induces partial downregulation of the activation pathway, both through autophosphorylation [26] and homologous desensitization of the receptor [27]. Thus, expression of eGFP-tagged PKC Apl II actually reduces the ability of 5HT to reverse synaptic depression by about 50% [11]. Generation of a new antibody to PKC Apl II with reduced background allowed for effective measurement of translocation of the endogenous PKC in sensory neurons and establishment of a dose response. In this case, instead of the normal post/pre ratio of translocation enabled by live imaging, the post-translocation ratio of PKC Apl II immunoreactivity was examined in sensory neurons and compared between different concentrations of 5HT. As the amount of translocation is quite variable, particularly between different batches of sensory neurons [28], translocation in each experiment was normalized to the amount of translocation measured with 10 μM 5HT, a saturating dose even when eGFP-PKC Apl II translocation was measured [15]. Approximately 50% of maximal translocation at 1μM was observed in these experiments (Fig 2A). Recently, we have observed significant differences in the desensitization of translocation after synapse formation and some differences in translocation parameters between synapses and cell bodies when synapses are formed [29]. To determine if this affected the requirement for 5HT, we compared translocation in cell bodies and processes in isolated sensory neurons and sensory neurons making synapses with motor neurons. We found a significant increase in the ability of 1 μM serotonin to translocate endogenous PKC Apl II in processes compared to the cell soma only after synapse formation (Fig 2B). This appears to be due to an increase in the relative ability of 5HT 1 μM to translocate PKC in the processes of synaptically paired sensory neurons as the difference between the relative amount of translocation in the processes between paired and unpaired neurons was significant with a P value of 0.04.

### Testing for involvement of different tyrosine kinases in PKC Apl II translocation

**Fig 3:**
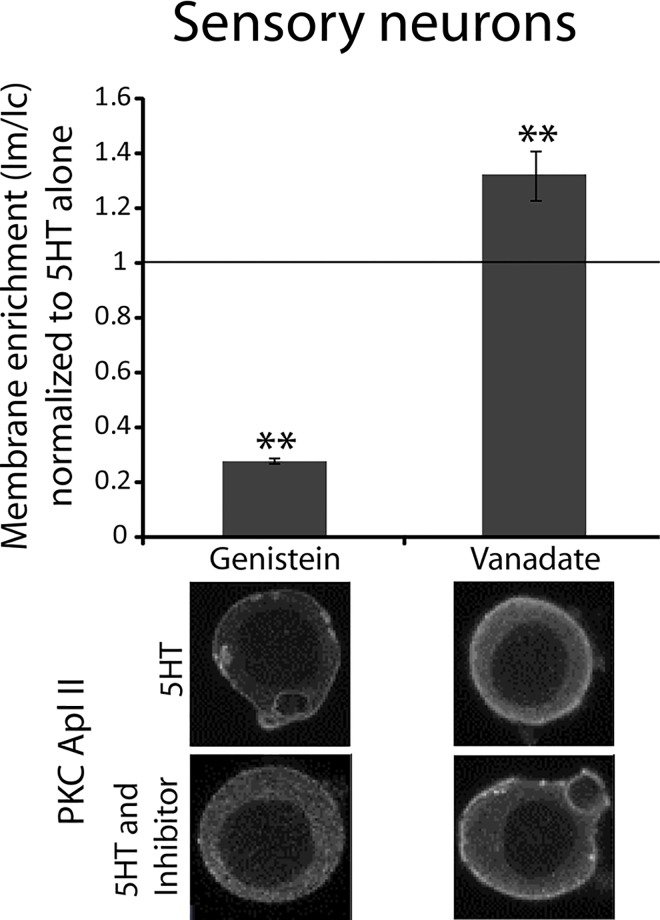
Genistein acts on a tyrosine kinase to inhibit endogenous PKC Apl II translocation in response to 5HT in isolated sensory neurons. The top panel shows the quantification of PKC Apl II membrane enrichment in the cell body of isolated sensory neurons (shown in the bottom panel) pretreated with either genistein (n = 19) for 5 min or vanadate (n = 26) for 30 min and then treated with 5HT (10 μM) for 5 min in the presence of the inhibitor. Cells were fixed and processed for immunocytochemistry using an anti-PKC Apl II antibody as described in methods. Membrane enrichment ratios were normalized to the 5HT alone group from the same experiments. The number of cells in the 5HT alone group for genistein and vanadate was 4 and 19 respectively. An unpaired Student’s t test was performed to compare normalized membrane enrichment in the 5HT and inhibitor group to that in the 5HT group, **p<0.01 after correction for multiple t-tests with Bonferroni correction. Error bars indicate SEM.

We have previously shown that genistein, a broad spectrum tyrosine kinase inhibitor, blocks both the 5HT-induced translocation of eGFP-PKC Apl II in isolated sensory neurons and reversal of synaptic depression [1]. We first confirmed that genistein blocks 5HT-mediated translocation of endogenous PKC Apl II. Cells were pretreated with genistein followed by 5HT in the presence of genistein as described in Methods. Cells were then fixed and processed for immunocytochemistry. As shown in Fig 3, cells treated with genistein showed significantly less membrane enrichment of PKC Apl II compared to cells treated with vehicle (the membrane enrichment ratio represents the translocation ratio in the genistein group normalized to the translocation ratio in the vehicle group). To confirm the involvement of tyrosine kinase signalling in PKC Apl II activation, we tested the effect of the tyrosine phosphatase inhibitor vanadate, on PKC Apl II translocation. As expected, vanadate had the opposite effect to that of genistein and increased 5HT-mediated translocation in cultured sensory neurons (Fig 3). Cells treated with the inhibitor alone did not show PKC Apl II membrane enrichment. Taken together, the above results confirm the involvement of tyrosine kinase phosphorylation in 5HT-mediated PKC Apl II activation.

**Fig 4:**
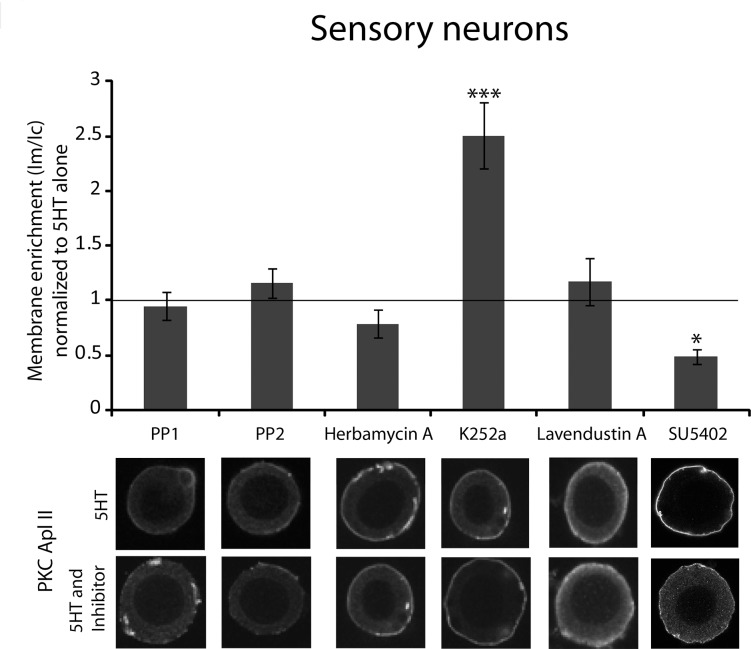
Testing the effect of different tyrosine kinase inhibitors on endogenous PKC Apl II translocation in response to 5HT in isolated sensory neurons. The top panel shows the quantification of PKC Apl II membrane enrichment in the cell body of isolated sensory neurons (shown in the bottom panel) pretreated with either PP1 (n = 14) or PP2 (n = 13) for 5 min, herbamycin A (n = 12) for 15 min, K252a (n = 39) for 5min, lavendustin A (n = 6) for 15 min or SU5402 (n = 17) for 60 min and then treated with 5HT (10 μM) for 5 min in the presence of the inhibitor. Cells were fixed and processed for immunocytochemistry using an anti-PKC Apl II antibody as described in methods. Membrane enrichment ratios were normalized to the 5HT alone group from the same experiments. The number of cells in the 5HT alone group for PP1, PP2, herbamycin A, K252a, lavendustin A and SU5402 was 17, 17, 23, 30, 19 and 18 respectively. An unpaired Student’s t test was performed to compare normalized membrane enrichment in the 5HT and inhibitor group to that in the 5HT group, ***p<0.001, *p = 0.06 after Bonferroni correction. Error bars indicate SEM.

To investigate the identity of the tyrosine kinase(s) involved, we tested the effect of inhibiting different tyrosine kinases on PKC Apl II translocation in response to serotonin. Sensory neurons were pretreated with the various inhibitors followed by 5HT (10 μM) treatment in the presence of the inhibitor as described above. We first tried PP1 and PP2, both inhibitors of the non-receptor tyrosine kinase Src family [30]. PP2 had previously been effectively used by our lab to block a likely tyrosine kinase phosphorylation-mediated inhibition of calcium currents in *Aplysia* [31]. As shown in Fig 4, neither PP1 nor PP2 inhibited membrane enrichment of PKC Apl II. We next tested herbamycin A, an antibiotic that selectively inactivates cytoplasmic tyrosine kinases [32] and was shown to block tyrosine kinase mediated activation of PLC [33] and PDGF-induced activation of PLD [34]. Herbamycin A had no effect on 5HT-induced translocation of PKC Apl II (Fig 4). Next, we examined inhibitors of receptor tyrosine kinases (RTKs) as one possibility is the transactivation of RTKs by G protein-coupled receptors (GPCRs). We tested K252a, a Trk family adenosine triphosphate (ATP)-based inhibitor previously shown to inhibit both *Aplysia* Trk and *Aplysia* Trk-like receptor [35,36]. K252a actually increased membrane enrichment of PKC Apl II compared to vehicle (Fig 4). This is presumably due to the fact that K252a is also known to inhibit PKCs directly [37] and as previously mentioned, PKC Apl II activity inhibits its own translocation [26,27]. Next, we tested lavendustin A, an epidermal growth factor receptor (EGFR) inhibitor previously used in *Aplysia* to study growth cone dynamics [38]. Lavendustin A did not affect membrane enrichment of PKC Apl II (Fig 4). Lastly, we tested SU-5402, a specific inhibitor of FGFR1 that requires interaction with Glycine (Gly) and Asparagine (Asn) residues in the ATP binding site of mammalian FGFR1 [39]. These residues are conserved in the *Aplysia* FGFR (S4 Table) As shown in Fig 4, SU-5402 inhibited membrane enrichment of PKC Apl II compared to vehicle suggesting a role for FGFR1 in PKC Apl II activation (Fig 4). In all experiments described with kinase inhibitors, treatment with the inhibitor alone did not affect basal PKC Apl II translocation.

### Testing for a role for FGF receptor in translocation of PKC Apl II

**Fig 5:**
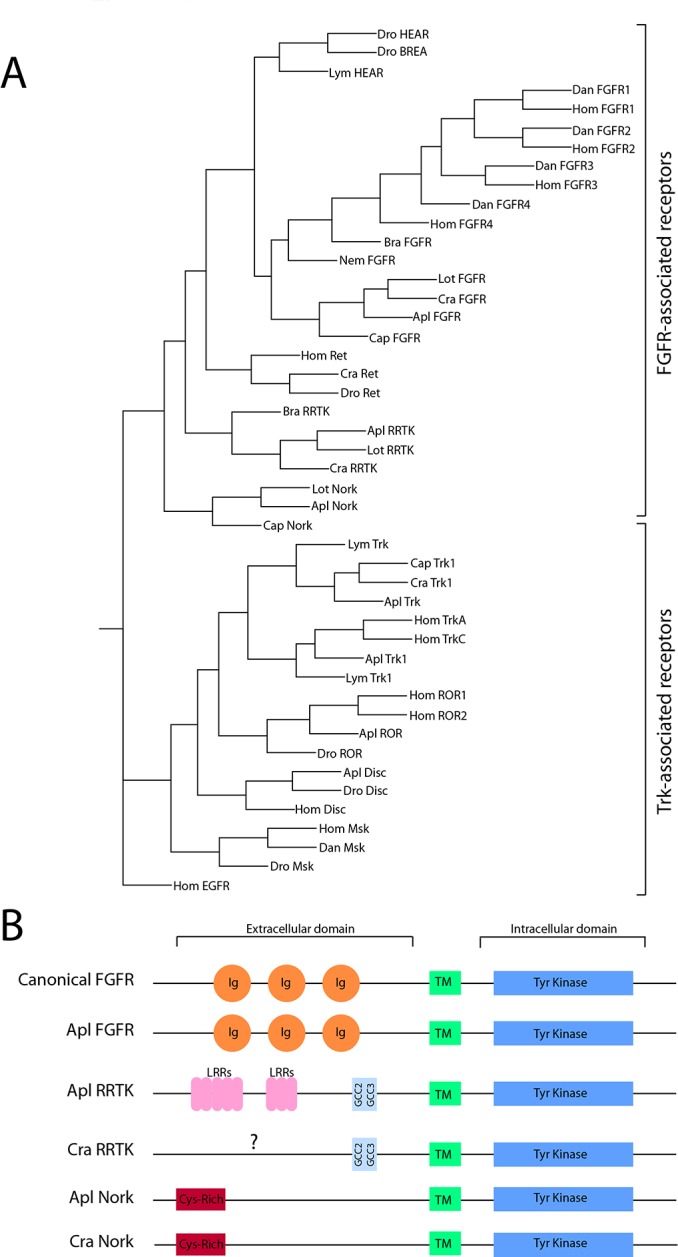
Phylogenetic analysis and structure of FGF-associated receptors. **A)** Phylogenetic analysis of selected FGF-associated receptors and Trk-associated receptors from invertebrates and vertebrates. The abbreviations are: Dan (Danio), Lot (Lottia), Apl (Aplysia), Lym (Lymnaea), Dro (Drosophila), Cap (Capitella), Cra (Crassostrea), Hom (Homo sapiens), Bra (Branchiostoma), Nem (Nematostella). The accession numbers are listed in Table 4. **B)** The extracellular domain of the Apl FGFR contains all the conserved domains associated with FGFRs, while neither Apl Nork nor Apl RRTK contains these domains. The abbreviations are: Ig, Immunoglobulin-like domain; Tyr Kinase, Tyrosine Kinase domain; LRRs, Leucine-Rich Repeats; TM, transmembrane domain; Cys-Rich, Cysteine-Rich region.

The above results suggest that serotonergic receptors may be transactivating FGFR1 to induce translocation of PKC Apl II. To investigate a role for FGFR1 in PKC Apl II activation, we cloned the *Aplysia* FGF receptor (Apl FGFR) and generated a mRFP-tagged version as described in Methods. There have been a number of reports of cloning of *Aplysia* FGFR-like receptors. FGF receptors are most closely linked to RET receptors and represent a separate superfamily of RTKs [40]. To determine the phylogenetic relationship between these receptors, we performed a Phylip analysis of the receptors whose kinase domain has been proposed to be in the FGFR family: *Aplysia* neurite outgrowth receptor kinase (ApNork) [41] and *Aplysia* leucine rich repeat receptor tyrosine kinase (Apl LRRTK) [42], with a subset of FGFR, Trk family and Ret receptor from the three major subdivisions of bilaterians. The results clearly show that the newly cloned Apl FGFR is the orthologue of the FGFR family in *Aplysia* (100% bootstrap probability for inclusion of Apl FGFR in the FGFR family as opposed to RET or TRK receptors) and that similarly to most invertebrates, there appears to be only one FGFR, with the four receptors in vertebrates due to a recent duplication (Fig 5A). Similarly, the two FGFRs in *Drosophila*, heartless and breathless, also represent a recent duplication. Apl Nork receptor appears to be present only in the Lophotrochozoa (mollusk and annelids), and while more similar to FGFRs than the Trk superfamily, is not more similar to FGFRs than to RET receptors. The recently cloned Apl LRRTK receptor, while called an FGFR-like receptor, is also more associated with the FGFR/RET family than the Trk superfamily, but similar to Apl Nork and unlike Apl FGFR, it is not more similar to the FGFR family than it is to the RET family. Using the kinase domain, there are orthologues of Apl LRRTK both in other mollusks and surprisingly in a primitive deuterostome, *Brachiostoma*. However, none of these orthologues share any extracellular domain homology with Apl LRRTK, suggesting the possible recent association of this extracellular domain with the intracellular tyrosine kinase domain. Similarly, the extracellular domain of the Apl FGFR contains all the conserved domains associated with FGFRs (Fig 5B), while neither Apl Nork nor Apl LRRTK contains these domains.

**Fig 6:**
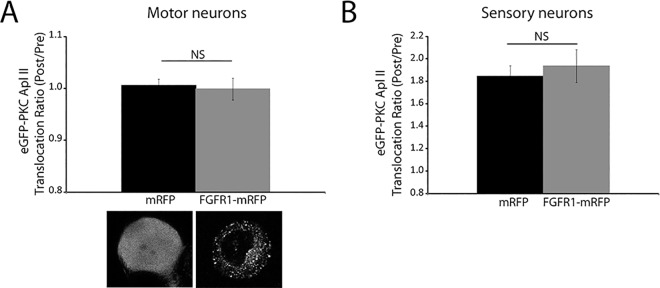
Overexpressing the FGF receptor in isolated motor or sensory neurons from *Aplysia* does not increase PKC Apl II translocation in response to 5HT. **A)** and **B)** eGFP-PKC Apl II was coexpressed along with either mRFP or FGFR1-mRFP in isolated motor neurons (A) or isolated sensory neurons (B) from *Aplysia* as described in methods. The number of cells in the mRFP group was 11 for the motor neurons and 19 for sensory neurons. The number of cells in the FGFR1-mRFP group was 7 for the motor neurons and 10 for sensory neurons. Cells were treated with 5HT (10 μM) for 5 min and PKC Apl II translocation ratio (Post/Pre treatment) was quantified. The bar graph shows the average of the translocation ratios measured 5 min after the addition of 5HT to the dish normalized to the Pre 5HT group. An unpaired Student’s t test was performed to compare eGFP-PKC Apl II translocation ratio in the FGFR1-mRFP group to that in the mRFP group in motor neurons (A) and sensory neurons (B). NS, non-significant; Error bars indicate SEM.

eGFP-PKC Apl II translocates in response to 5HT in sensory neurons and not motor neurons [15], thus if FGFR represented a key step in PKC Apl II translocation, expression in motor neurons should allow translocation. Despite observation of mRFP staining on membranes of motor neurons after expression (Fig 6A), expression of the *Aplysia* orthologue of FGFR did not allow PKC Apl II translocation in motor neurons (Fig 6A) nor did it affect PKC Apl II translocation in sensory neurons, as might be expected if the FGFR was the rate limiting step in PKC Apl II transloction (Fig 6B).

### Testing for a role for B receptors in translocation of PKC Apl II

**Fig 7:**
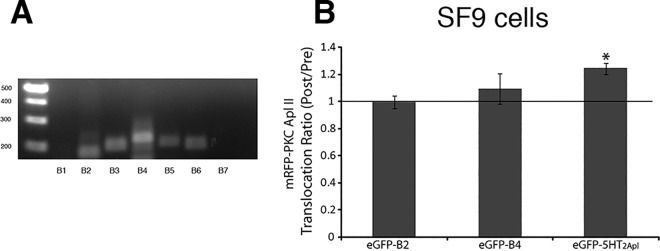
B receptors do not contribute to PKC Apl II translocation downstream of 5HT in Sf9 cells. **A)** RT-PCR analysis of B receptors in *Aplysia* central nervous system (CNS). B2, B3, B4, B5 and B6 receptors were expressed in CNS, whereas B1 and B7 receptors were not. **B)** Sf9 cells were cotransfected with mRFP-PKC Apl II along with either eGFP-B2 (n = 3), eGFP-B4 (n = 3) or eGFP-5HT_2Apl_ (n = 3). Cells were treated with 5HT (10 μM) for 5 min and PKC Apl II translocation ratio (Post/Pre treatment) was quantified as described in methods. The histogram shows the average of the translocation ratios measured at 60, 90, 120 and 150 sec after the addition of 5HT to the dish normalized to the Pre 5HT group. A paired Student’s t test was used to compare translocation post 5HT to pre 5HT for each group, *p<0.05 after correcting for multiple tests with Bonferroni correction. Error bars indicate SEM.

Two “B” receptors were initially identified as putative 5HT receptors, although the data indicating binding to 5HT in an exogenous cell line was later explained by endogenous 5HT receptors present in this line [43]. Phylogenetic analysis suggested that B receptors probably derived from the *Aplysia* D1 receptor, but have undergone considerable changes since this point, such that the *Aplysia* D1 receptor is closer to vertebrate D1 receptors than to the diverged B receptors [1]. While two receptors were initially identified, examination of the *Aplysia* genome shows the presence of seven receptors, and many of them are on genomic loci with consecutive number suggesting that they are on the same genomic region, probably the result of gene duplication (S5 Table). A PCR screen of nervous system cDNA demonstrated that most of the receptors are expressed in the nervous system (Fig 7A). We cloned two of the receptors, including the original B2 receptor [43], and generated green fluorescent protein (eGFP)-tagged B2 and B4 receptors as described in Methods and co-expressed each with monomeric red fluorescent protein (mRFP)-PKC Apl II in Sf9 cells. Cells were treated with 5HT (10 μM) for 5min. As shown in Fig 7B, neither eGFP-B2, nor eGFP-B4 co-expression induced 5-HT mediated translocation of PKC Apl II in Sf9 cells, despite the ability of the 5HT_2Apl_ receptor to stimulate translocation in these cells (Fig 7B).

## Discussion

### Using endogenous PKC Apl II translocation to monitor PKC Apl II activation

Most of our studies on translocation have used live imaging of fluorescently tagged PKCs [16,21,26]. Here, we complement this assay with measuring translocation of the native PKC Apl II with immunocytochemistry. There are advantages and disadvantages of both assays. Using live imaging, one has an image of the state of translocation before 5HT addition and thus can measure translocation in a sensitive manner using a Post/Pre measurement. However, this requires overexpression of the tagged PKC and desensitizes the response. Reversal of depression was decreased by 50% when PKC Apl II was overexpressed indicative of this desensitization [11]. The desensitization is partly due to autophosphorylation of the C2 domain strengthening the normal inhibitory role of this domain [26] and partly due to homologous desensitization of the signalling pathway between 5HT and PKC Apl II [27]. These are probably not only important for the overexpressed PKC since we see enhanced translocation of the endogenous PKC Apl II with K252a, which inhibits PKC, as well as Trk receptors. While anecdotally there appears to be larger translocation of the endogenous PKC Apl II than eGFP-tagged PKC Apl II, large variability in translocation between individual cells makes it difficult to establish this as a significant difference. This is one disadvantage for measurements of the endogenous kinase, as many more cells need to be measured to account for the increased variability.

There has not been a previous dose response for 5HT of PKC Apl II translocation. Our results show approximately half-maximal activation in both isolated sensory neurons and sensory neurons co-cultured with motor neurons at 1 μM. The expected maximum concentration of 5HT released physiologically is approximately 100 nM [7], although concentrations may be higher if these bulk measurements made using carbon fibers underestimate the concentration reached at terminals. Interestingly, while there have been some indications that low concentrations of 5HT preferentially activate reversal of depression vs facilitation [25], short-term facilitation caused by PKA saturates at 100 nM 5HT in cultures [44]. Thus, it appears that, at least in cultured neurons this pathway is more sensitive to low concentrations of 5HT. One possibility for the discrepancy is that the receptor complex important for activation of PKC Apl II in ganglia is different and more sensitive to 5HT than the complex present in cultured neurons. Translocation measures the increased affinity of PKC for the lipid phospatidylserine in the presence of its lipid activators (diacylyglycerol and phosphatidic acid) [16]. At low concentrations of these activators, there may be preferential activation of a pre-localized pool of PKC Apl II and this would not observed in our assay since this pool of PKC may already appear to be membrane localized in live imaging or in imunocytochemistry. Indeed, prevention of synaptic depression is mediated by a pool of activated PKC Apl I that is not easily detected in translocation assays [45].

### Our results confirm an important role for tyrosine phosphorylation in the translocation of PKC Apl II

Our results confirm the requirement for 5HT activation of a tyrosine kinase for PKC Apl II translocation. However, while inhibitors of FGFR slightly decreased translocation, the FGFR is not the missing component that would allow 5HT translocation in motor neurons. One possibility is that the FGFR plays a small facilitatory role as opposed to being the major trans-activated tyrosine kinase. Another possibility is that the motor neurons might lack some other components of an FGFR intracellular signaling cascade which are required to link FGFR expression to PKC Apl II translocation. Moreover, SU-5402 is also a potent inhibitor of the vascular endothelial growth factor (VEGF) receptor thus the VEGF receptor might be the missing component for PKC Apl II translocation in motor neurons. The VEGF receptor has not been cloned yet from *Aplysia* but our bioinformatic analysis confirmed that the asparagine to which SU-5402 binds in the ATP binding site is conserved in the *Aplysia* VEGF receptor sequence (S4 Table). While there was no effect of expressing the FGFR in sensory neurons, the levels of the receptor may not be rate-limiting. Thus, we cannot rule out the possibility that it plays an important role in these neurons without examining PKC Apl II translocation in the absence of the receptor. Using a siRNA knockout approach would require an antibody to the *Aplysia* FGFR, which we do not have at this point. Our results also rule out a number of other candidates including Src family members EGF receptors and Trk kinases. However, there are a large number of additional RTKs in the *Aplysia* genome that have not yet been characterized, although there is no obvious candidate at this time.

### What is the mechanism for 5HT-mediated activation of the novel PKC Apl II?

Regardless of the tyrosine kinase required for PKC Apl II translocation, there still needs to be a transmembrane G protein-coupled receptor that binds 5HT and is required for PKC Apl II translocation. Despite the pharmacological evidence that the cloned 5HT_2Apl_ receptor is blocked by pirenperone, but neither reversal of depression, nor PKC Apl II translocation is blocked by pirenperone, it is difficult to rule out this receptor. First, pharmacological agents can have distinct effects on different downstream targets, and the mechanism for activation in Sf9 cells may be different from in *Aplysia* neurons. Indeed, activation of 5HT_7Apl_ can lead to translocation of PKC Apl II in Sf9 cells, but not in neurons and we show here this is likely independent of adenylate cyclase. Thus, translocation in Sf9 cells may not be the best model system to examine which receptors in *Aplysia* are important. Second, the pirenperone sensitive receptor expressed in Sf9 cells is not necessarily the same as the receptor expressed in *Aplysia* neurons as it was necessary to remove the long-N terminal extension of the receptor for expression in Sf9 cells or neurons (1). Moreover, we have evidence that the receptor complex changes after synapse formation [29], and this is not recapitulated in isolated sensory neurons. We have also been unable to verify knock down of the 5HT_2Apl_ receptor with antibodies and antisense oligonucleotides we have generated to the receptor (unpublished data). One possibility is that there is an additional 5HT2 receptor in *Aplysia*. In their recent publication of the *Hermissenda* transcriptome, Katz and colleagues identified a 5HT2 receptor with more similarity to arthropod 5HT_2B_ receptors than to the 5HT_2Apl_ receptor we had cloned [46]. The NCBI database reveals a receptor that may be the orthologue of this *Hermissenda* receptor (XP_012935429.1) in *Aplysia*.

In summary, the GPCR and RTK receptors important for PKC Apl II translocation are still unknown.

## Supporting Information

S1 Table List of Primers used for cloning of the Aplysia B receptors. (PDF)

S2 Table List of primers used for cloning of the *Aplysia* FGF receptor. (PDF)

S3 Table List of primers used to transfer the FGFR1-like segments into the *Aplysia* expression vector pNEX3. (PDF)

S4 Table The target residues for the FGFR inhibitor SU-5402 are conserved in Aplysia. All of the receptors used in the bioinformatics evaluation of FGF receptors are shown with the 9 amino acid region important for binding to SU-5402 shown. The conserved alanine (A) and asparagine (N) are shaded in red when present. Accession numbers are given for all receptors.
(PDF)

S5 Table Genomic accession numbers for B receptors. The genomic locus and the nucleotides in the locus encoding the B receptor isoform are shown. It is also indicated when the entire B receptor sequence is not present in this locus.
(PDF)

S1 File Supporting data excel file. All data that make up the figures is stored in this file as pre PLOS One requirements.
(XLSX)
