# Supplementary material for: Investigating the Potential Signaling Pathways That Regulate Activation of the Novel PKC Downstream of Serotonin in Aplysia
Source: PLoS One. 2016 Dec 21;11(12):e0168411. doi: 10.1371/journal.pone.0168411 (PMC5176290; doi:10.1371/journal.pone.0168411)
Supplement: S1 Table — (PDF) [file pone.0168411.s001.pdf]

S1 Table. List of PCR primers for cloning of the Aplysia B receptors.

**B1 F**	GAGTGGCATGAACTTTCAATG
**B1 R**	CTTGATCCGGACGTGATGT
**B2 F**	GAAGCCACTACAGTACGAGGA
**B2 R**	GTTTGATGTCCGGTTATCTC
**B3 F**	AGTGGCATGAGCTCTCAGTG
**B3 R**	CTTGACCTCTAAGCTGTGGA
**B4 F**	GATGTCTGCAGACGTCATGTG
**B4 R**	GACCGTGTCTTTGTATGGA
**B5 F**	GATGTCTGCAGACGTGATGTG
**B5 R**	AACGTCTGTGCTTCAGCAG
**B6 F**	TCATCTGGATGTCTGCAGAC
**B6 R**	TGTGTGCTTCATGGGCTTC
**B7 F**	ACGACCTGTACGGTTACTG
**B7 R**	CTTCCACTGAGAGCTCATG
