# Supplementary material for: Investigating the Potential Signaling Pathways That Regulate Activation of the Novel PKC Downstream of Serotonin in Aplysia
Source: PLoS One. 2016 Dec 21;11(12):e0168411. doi: 10.1371/journal.pone.0168411 (PMC5176290; doi:10.1371/journal.pone.0168411)
Supplement: S2 Table — (PDF) [file pone.0168411.s002.pdf]

S2 Table. List of PCR primers used for cloning of the *Aplysia* FGF receptor

**Segment 1 Outer F**	CCGCCGCATTAGCTCCTCTA
**Segment 1 Outer R**	GCATTCTGCACCAGACAAGT
**Segment 1 Inner F**	GATGGGATATTACTGGTCGC
**Segment 1 Inner R**	TGCTGTCATCAATTGTGCCC
**Segment 2 F**	ATCCAGGTGAGCGACACCCA
**Segment 2 R**	CCATTTCACGGATGAGGTCC
**Segment 3 Outer F**	GTGATTGTGGTGAGCACGGCC
**Segment 3 Outer R**	AGCCGGACGCACACATGACCCTA
**Segment 3 Inner F**	GACACCACGGAGCTCTCCGAGTAC
**Segment 3 Inner R**	CTCAATGACAGAGTTATCCCCGGAGCT
