# Supplementary material for: Investigating the Potential Signaling Pathways That Regulate Activation of the Novel PKC Downstream of Serotonin in Aplysia
Source: PLoS One. 2016 Dec 21;11(12):e0168411. doi: 10.1371/journal.pone.0168411 (PMC5176290; doi:10.1371/journal.pone.0168411)
Supplement: S3 Table — (PDF) [file pone.0168411.s003.pdf]

S3 Table. List of primers used to transfer *Aplysia* FGFR1-like segments into the *Aplysia* expression vector mRFP-pNEX3

**Segment 1 F**	GCGCAGGTCGACTCTAGCGCTACCGGTCGCCACCGATGGGATATTACTGGTC
**Segment 1 R**	GCGCCTCCTTGATGACGTCCTCGGAGGAGGCCATGCTACAGGAGAAGATGG
**Segment 2 F**	GCGCGTCGGGGAGACGGCCATCTTCTCCTGTAGCATCCAGGTGAGCGACACC
**Segment 2 R**	GCGCCTCCTTGATGACGTCCTCGGAGGAGGCCATCATTTCACGGATGAGGTC
**Segment 3 F**	GCGCAGAGAAATGATGGACCTCATCCGTGAAATGGAGACGATGAAACTCATC
**Segment 3 R**	GCGCCTCCTTGATGACGTCCTCGGAGGAGGCCATCTCAATGACAGAGTTATC
