# Supplementary material for: Investigating the Potential Signaling Pathways That Regulate Activation of the Novel PKC Downstream of Serotonin in Aplysia
Source: PLoS One. 2016 Dec 21;11(12):e0168411. doi: 10.1371/journal.pone.0168411 (PMC5176290; doi:10.1371/journal.pone.0168411)
Supplement: S4 Table — All of the receptors used in the bioinformatics evaluation of FGF receptors are shown with the 9 amino acid region important for binding to SU-5402 shown. The conserved alanine (A) and asparagine (N) are shaded in red when present. Accession numbers are given for all receptors. (PDF) [file pone.0168411.s004.pdf]

S4 Table. The target residues for the FGFR inhibitor SU-5402 are conserved in *Aplysia*

AplFGFR	FAPHGNLRD	XP_012942529.1
CraFGFR	FAPHGNLRD	XP_011422739.1
LotFGFR	YAPHGNLRD	XP_009056169.1
CapFGFR	YAPHGNLRD	ELT91660.1
DanFGFR1	FAAKGNLRE	XP_009302579.1
HomFGFR1	YASKGNLRE	AAA35835.1
DanFGFR2	YASKGNLRE	CAM73170.1
HomFGFR2	YASKGNLRE	AAA52449.1
DanFGFR3	YASKGNLRE	CAM73234.1
HomFGFR3	YAAKGNLRE	AAI21176.1
DanFGFR4	YASKGSLRE	AAA96816.1
HomFGFR4	CAAKGNLRE	ABQ01235.1
BraFGFR	YAEHGNLRD	ABD24302.1
NemFGFR	YASQGNLRQ	ABO92762.1
LimFGFR	YAPNGNLRE	XP_002431203.1
DroBREA	YAPHGNLKD	NP_729956.1
DroHEAR	YAPHGNLKD	NP_732286.1
AplVEGF	YCHFGNLRT	XP_012946495.1
HomVEGF3	YCKYGNLSN	P17948.2
HomTrkA	YMRHGDLNR	BAA34355.1
HomTrkC	YMKHGDLNK	CAA12029.1
LimTrk	YMELGDLNN	XP_013787385.1
AplTrk	FMEHGDLNK	NP_001232923.1
CraTrkl	YMIHGDLAE	EKC18559.1
CapTrkl	YMEHGDLTE	ELT97150.1
AplTrkl	YMEHGDLSE	NP_001191591.1
LimTrkl	YMQYGDLAE	XP_013776854.1
AplRor	YMAQGDLHE	AAK25726.1
DroRor	YMANGDLHE	NP_476962.1
HomRor1	YINQGDLHE	Q01973.2
HomROR2	YCSHGDLHE	Q01974.2
DanMSK	YMAYGDLNE	NP_001004503.1
HumMsk	YMAYGDLNE	NP_001159752.1
DroMsk	YMAPGDLSE	BAA20134.1
CraRet	YCEHGSLLQ	EKC27750.1
Homret	YAKYGSLRG	P07949.3
DroRet	YARYGSLRS	NP_477044.1
APlNork	YVPYGNLQN	AAP47187.1
LotNork	FIPHGNLQT	XP_009055745.1
CapNork	YMPFGNLQN	ELT89841.1
AplRRTK	YAELGDLLS	NP_001232922.1
LotRRTK	LAELGDLLT	XP_009049757.1
CraRRTK	FASNGDLLS	XP_011437316.1
BRaRRTK	YAMHGNLRE	XP_002601791.1
DroDisc	YHCLGDLNQ	NP_001014474.3
AplDisc	YMKYGDLNQ	XP_012945560.1
HomDisc	YMENGDLNQ	Q08345.1
HomEGFR	LMPYGCLLD	P00533.2
