# Supplementary material for: Investigating the Potential Signaling Pathways That Regulate Activation of the Novel PKC Downstream of Serotonin in Aplysia
Source: PLoS One. 2016 Dec 21;11(12):e0168411. doi: 10.1371/journal.pone.0168411 (PMC5176290; doi:10.1371/journal.pone.0168411)
Supplement: S5 Table — The genomic locus and the nucleotides in the locus encoding the B receptor isoform are shown. It is also indicated when the entire B receptor sequence is not present in this locus. (PDF) [file pone.0168411.s005.pdf]

S5 Table. Genomics accession numbers for B receptors

B receptor	Genomic locus	Nucleotides
AplB1	AASC03097936.1	1-890 (incomplete)
AplB2	?	
AplB3	AASC03097938.1	2842-4102
AplB4	AASC03097943.1	1814-2975
AplB5	AASC03097942.1	3845-5010
AplB6	AASC03097937.1	3708-4852
AplB7	AASC03138538.1	1291-642 (incomplete)
